# Medical News and Miscellany

**Published:** 1889-05

**Authors:** 


### Medical News and Miscellany.

The biggest surgical undertaking on record: Lanc(s)ing Michigan!

Dr. J. W. Garnett, of Greenville, Hunt county, is attending a course at the N. Y. Polyclinic.

Important notice to members of the T. S. M. A.: Members who read papers at the recent meeting, and who have retained them for revision are notified that the papers *must be* in the hands of the Publishing Committee prior to June 1st or they will be excluded from the Transactions.

DR. E. T. COOK, of Trinity, will leave shortly to attend the New York Polyclinic.

---

MARRIED in Crystal Springs, Miss., May——inst.: Dr. J. H. Rucker, of Holland, Texas, to Miss Imogene Bailey, of the former city.

---

FOR SALE.—A complete set of "International Encyclopedia of Surgery," (by Ashhurst *et at.*,) 6 volumes, in ½ Morocco; cost $48; in as good condition as when issued. The owner will sell it for $38, which is over 20 per cent. discount. Address the editor of this journal. A rare opportunity.

---

How does this number of the JOURNAL strike you, Doctor? Is such a publication not deserving of cordial support, by every member of the State Association, at least? We think so. Only $2 a year, is asked for it. Twenty-four extra pages and a costly portrait. Show it to your neighbor and get him to send in his subscription.

---

DR. R. L. GRANT, representing the Dios Chemical Co., of St. Louis, and Chambers' puplications, the Weekly Review and Annals of Surgery, favored the JOURNAL with a call, impressing us most favorably as a live, active and intelligent agent. Dr. G. will make a thorough canvass of the State, and we bespeak for him a courteous reception by the doctors.

---

THE MEDICAL BRANCH TEXAS UNIVERSITY.—The City Council of Galvestion have appropriated $25,000 towards building the Texas Medical College, (Medical Department State University.) The State, at last session of the Legislature, appropriated $50,000, and the site has beeu donated by private citizens; so the prospect is that we will soon have a medical school under State management.

---

THE NEW ORLEANS MEDICAL AND SURGICAL JOURNAL in speaking of the recent meeting of the Louisiana State Medical

Association, says: "If we are ever to equal the Texas or Alabama Associations, we are to hunt out our errors and pluck them out."

A few years ago, before the publication of DANIEL'S TEXAS MEDICAL JOURNAL was begun, few had heard of the Texas State Association, outside of the State; now it is cited as a model for emulation—good.

---

OWING to the full report of proceedings Texas State Medical Association in this issue, there is not room for the usual amount of News and Miscellany and Book Notices, and we are compelled to omit much interesting matter. But, these "big things" only come, like Christmas, once a year. We only regret that we did not have the room to get in some account of the banquet and the Corpus Christi excursion, for, as the Queen of Sheba exclaimed on one occasion, "the half has not been told!"

---

THE American Medical Association will hold its fortieth annual session at Newport, R. I., on Tuesday, Wednesday, Thursday and Friday, June 25, 26, 27, 28, prox. Dr. W. B. Atkinson, Philadelphia, Secretary.

"Each State, county and district medical society entitled to representation, shall have the privilege of sending to the association one delegate for every ten of its regular resident members and one for every additional fraction of more than half that number."

The following are the delegates appointed by the Texas State Medical Association at San Antonio: Drs. O. Eastland, A. W. Pope, B. W. Bristow, J. R. Briggs, F. E. Yoakum, W. T. Jones, J. Van Gaskin, I. E. Clarke, T. J. Wagley, F. C. Ford, J. W. Carhart, J. W. Garnett, and J. H. Sears. It will be seen that only twelve were appointed, whereas the association is entitled to fifty delegates—there being now 502 members. If there are other members of the association who would like to attend the Newport meeting, certificates will be issued them upon application to the secretary, Dr. F. E. Daniel, who was authorized to complete the list, giving certificates only to those who desire and intend to attend the convention.

---

THE NORTH TEXAS MEDICAL ASSOCIATION is one of the foremost medical organizations in the State, in point of numbers and scientific work. Dr. J. T. Wilson, of Sherman, is President, and Dr. G. R. Clayton, Secretary.

The next semi-annual meeting will be held in Paris—beginning Tuesday, June 11, and holding three days. We have received the programme and a courteous invitation to be present, and much regret our inability to do so. Papers will be presented by Drs. Bacon Saunders, J. H. Smith, W. F. T. Savage, J. F. Hooks, S. F. King, R. R. Walker, V. A. Howeth, F. E. Yoakum, and T. G. Bates.

---

GALVESTON'S SEMI-CENTENNIAL.—Galveston is putting her best foot foremost in an effort to befittingly celebrate the semi-centennial anniversary of the incorporation of the city by the sea. Preparations for a grand civic and military display, probably unsurpassed in the history of the South, are being made. The fun will commence promptly and early on the 4th of June and continue until late in the night of June 15th. Reduced rates over all railroads from all points in the State will be secured, and those who want to bathe in the glorious surf and breathe the wave-kissed breezes from the Gulf of Mexico will not soon again be afforded such an opportunity. In addition to all the natural attractions, the management having charge of the arrangements announce that the military display on this occasion will be something marvelous. Altogether prizes aggregating $22,975 are offered for competing military, horse racing and regattas.

At night the grand panoramic spectacular military drama, "Fall of Paris and Reign of the Commune," will furnish a spectacle worth going hundreds of miles to see. This is one of the most startling and realistic productions ever witnessed in the South.

On the encampment grounds the regular army will be represented by four companies of infantry, six companies of cavalry, a battery and military band. Five interstate and twelve State companies have already entered, and it is safely estimated that there will be 5000 soldiers in camp. The camp grounds are within two miles of the heart of the city, and reached by the Galveston & Western Railroad, affording ample and cheap transportation. Galveston extends an invitation, especially to the people of Texas, to join with her in celebrating her semi-centennial.

